# Dimeric structure of p300/CBP associated factor

**DOI:** 10.1186/1472-6807-14-2

**Published:** 2014-01-14

**Authors:** Shasha Shi, Juanyu Lin, Yongfei Cai, Jiao Yu, Haiyan Hong, Kunmei Ji, Jennifer S Downey, Xiaodong Lu, Ruichuan Chen, Jiahuai Han, Aidong Han

**Affiliations:** 1 State Key Laboratory of Cellular Stress Biology, School of Life Sciences, Xiamen University, Xiamen 361102, China; 2 School of Medicine, Shenzhen University, Shenzhen, Guangdong 518060, China; 3 Division of Biomedical Science, Herman Ostrow School of Dentistry of University of Southern California, Los Angeles, CA 90089, USA; 4 Department of Biomedical Sciences, School of Life Sciences, Xiamen University, 3 S. Xiangan Road, Xiamen, Xiangan 361102, China

**Keywords:** PCAF, Histone acetyltransferase, Dimerization, ATAC

## Abstract

**Background:**

p300/CBP associating factor (PCAF, also known as KAT2B for lysine acetyltransferase 2B) is a catalytic subunit of megadalton metazoan complex ATAC (Ada-Two-A containing complex) for acetylation of histones. However, relatively little is known about the regulation of the enzymatic activity of PCAF.

**Results:**

Here we present two dimeric structures of the PCAF acetyltransferase (HAT) domain. These dimerizations are mediated by either four-helical hydrophobic interactions or a ß-sheet extension. Our chemical cross-linking experiments in combined with site-directed mutagenesis demonstrated that the PCAF HAT domain mainly forms a dimer in solution through one of the observed interfaces. The results of maltose binding protein (MBP)-pulldown, co-immunoprecipitation and multiangle static light scattering experiments further indicated that PCAF dimeric state is detectable and may possibly exist in vivo.

**Conclusions:**

Taken together, our structural and biochemical studies indicate that PCAF appears to be a dimer in its functional ATAC complex.

## Background

The unique posttranslational modification patterns on histones have been conceptualized as epigenetic codes that may finely tune transcription of specific genes [1,2]. Histone acetyltransferases (HATs), including p300/CBP and PCAF/GCN5, are responsible for modification of histones by acetylation on the exposed lysines [3-5].

PCAF/GCN5 are important members of histone acetyltransferases. Homozygous GCN5 knockout mice died during embryogenesis, while the majority of PCAF knockout mice developed normal [6,7]. However, the PCAF knockout mice later showed memory impairment, psychological anxiety and defects in stress control [8,9]. Interestingly, a single nucleotide polymorphism in the PCAF gene was found in patients with coronary heart abnormalities that resulted in vascular morbidity and mortality [10].

Metazoan PCAF/GCN5 proteins have three conserved domains–N terminal extension region, HAT domain and bromodomain (BRD) [11]. The HAT and BRD are also highly conserved in yeast and plants [12,13]. The HAT domain of PCAF/GCN5 has a globular structure that contains an acetyl-CoA binding pocket [14,15]. Acetyl-CoA binds the HAT domain through the pyrophosphate body and pantetheine arm [16,17]. Substrates of histones and non-histones, such as p53, induce large conformational changes in the active pocket of the PCAF HAT domain through extensive interactions that anchor specific lysines for acetylation [16,18]. Interestingly, PCAF/GCN5 have slightly different specificity. GCN5 acetylates histones H3 and H4 with favorable sites of lysines 9 and 14 on histone H3 and lysines 8 and 16 on histone H4 [19]. In comparison, PCAF mainly acetylates lysine 14 on H3 [20] and also specifically acetylates p53 at lysine 320 to enhance responses to DNA damage [21,22].

More importantly, the metazoan PCAF/GCN5 are usually present in the megadalton complexes SAGA (Spt-Ada-GCN5-acetyltransferase) and ATAC (Ada-Two-A containing complex) [11,23]. The additional components Ada2 and Ada3 (alteration/deficiency in activation) are required to form a minimal core complex that can efficiently acetylate histone octamer and nucleosome [24,25]. In addition to a PCAF binding domain, Ada2 has a SWIRM domain that binds nucleosomal DNA [26]. A SANT domain at Ada2 N terminus has been proposed to direct histone tails to specifically associate with PCAF catalytic site [27-29]. Through a structurally unknown C terminal domain Ada3 physically associates with Ada2 in the core complex [30]. However, the molecular mechanism of this core complex in the regulation of PCAF/GCN5 catalytic activity and specificity remains largely unknown.

In this report we present structure-based biochemical characterization of the PCAF HAT domain. We show that the PCAF HAT domain can form a dimer in a concentration dependent manner. All our experimental data suggest that PCAF may exist as a possible dimer in its ATAC complex.

## Results

### Overall structures

The PCAF HAT domain (amino acids 493-658) was purified and crystallized in a unique condition that contained 1.2 M ammonium sulfate and 0.2 M lithium sulfate. The structure was solved by molecular replacement. To our surprise, we found four HAT domains in one asymmetric unit (Additional file 1: Figure 1S). The structure of each HAT domain remains almost the same globular folding (root mean square deviation (rmsd) of 0.032-0.035 for Cα backbone). The overall structure is similar to the PCAF structure (rmsd of 0.397 for Cα backbone) solved by Marmorstein and his co-workers [17], except for the loop 1 (L1), which was not defined due to lack of clear electron density (Additional file 1: Figure S2A and S2B, indicated by arrow). Our PCAF structure is also similar to human GCN5 (rmsd of 0.34 for Cα backbone) [31] and *Tetrahymena* GCN5 (rmsd of 0.902 for Cα backbone) [16]. In addition to the L1, loop 2 (L2) is also significantly different from *Tetrahymena* GCN5, which is bound by a histone peptide (Additional file 1: Figure S1C and S1D, indicated by arrow).

**Figure 1:**
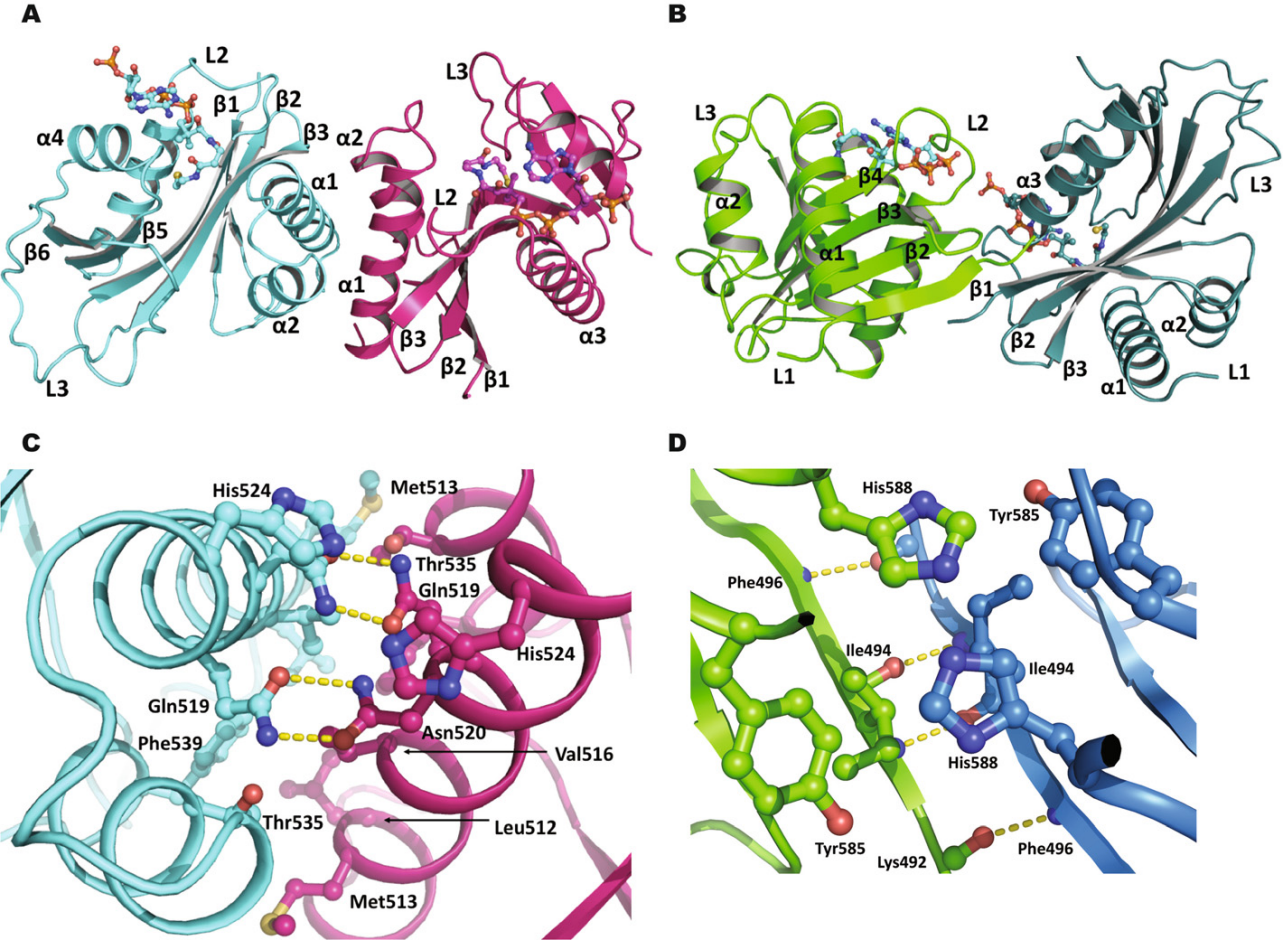
**Dimeric structures of the PCAF HAT domain. A)** The PCAF dimer structure mediated by a four helical bundle; **B)** The PCAF dimer structure mediated by β-sheet extension; **C)** Close-up view of detailed interactions within the PCAF dimeric interface shown in A; **D)** Close-up view of detailed interactions in the PCAF dimeric interface shown in B. The structures are presented in ribbon with each monomer colored in cyan, magenta, green and blue. Helices, β-strands and loops are labeled with α, β and L, respectively. The cofactor acetyl-CoA is represented in sticks **(A and B)**. Amino acids that are involved in direct contacts are represented as sticks and also labeled **(C and D)**. Hydrogen bonds are indicated with yellow broken lines.

We found that the four PCAF HAT domains in one asymmetric unit form two different dimers (Figure 1A and B). The residues of both interfaces were very well defined on electron density maps (Additional file 1: Figure 3S). The first dimer is formed by four-helical stacking of helices α1 and α2 (Figure 1A and C) and has a canonical interface with a buried surface area of 1530 Å^2^. The four residues Leu512, Val516, Thr535 and Phe539 from both monomers form a central hydrophobic core, while Gln519 and Asn520 create four pairs of hydrogen bonds. In addition, Met513 and Thr535 or two His524 residues located at the periphery of the interface make additional van der Waals contacts.

The second dimer of the PCAF HAT domain is mediated through the ß-sheet extension of two antiparallel ß1 strands (Figure 1B and D). The major contacts of this dimer are three hydrogen bonds between amino acids 493-495. This interface is further stabilized by a hydrogen bond from two His588 residues and van der Waals contacts from two Ile494 and Tyr585 and His588 from each monomer. The buried surface area of this interface is 879 Å^2^.

Taken together, the results show that the PCAF HAT domain forms two distinct dimers. The clusters of hydrophobic residues contribute their important roles to the interactions. While the first interface is significant, however, the second interface may likely be produced during crystal packing due to the smaller size and insignificant score calculated by PISA program (Additional file 1: Table S1).

### PCAF dimers detected by pulldown experiments

**Figure 2:**
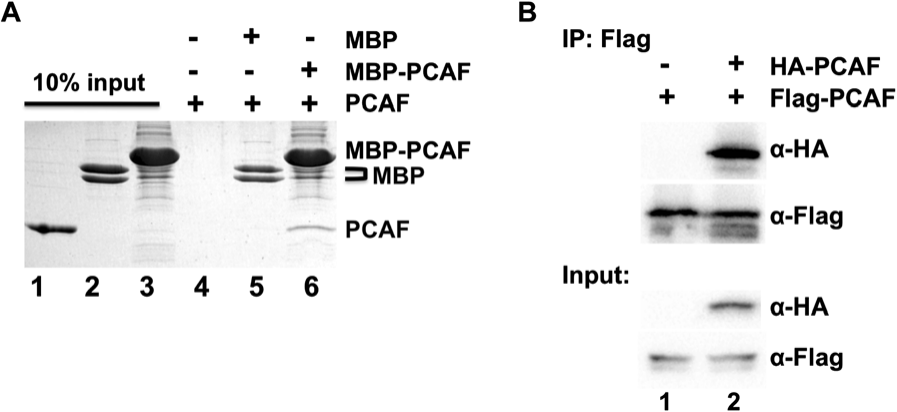
**PCAF HAT domain interacts with each other in solution. A)** MBP pulldown experiment using an MBP-PCAF HAT domain fusion. Purified MBP-PCAF fusion proteins were treated with 2 M NaCl to remove any bound maltose and mixed with new amylose beads and His-tagged PCAF fragments (lanes 4-6). The beads were then washed off unbound proteins and analyzed by 15% SDS-PAGE. Ten percent inputs of His-tagged PCAF fragments, MBP alone and MBP-PCAF fusion protein were used as loading controls (lanes 1-3). **B)** The presence of PCAF dimer state examined by co-immunoprecipitation. A plasmid that expresses Flag-PCAF was co-transfected with an empty vector pLV-nHA (lane 1) or HA-PCAF expression plasmid (lane 2). The expressions of Flag-or HA-PCAF were confirmed using 5% input shown at the bottom, while the Flag gel after wash was checked by western blotting as shown at the top.

We next wanted to know whether PCAF HAT domain exists as a dimer in solution. We first used a maltose binding protein (MBP) affinity pulldown experiment. The PCAF HAT domain in MBP fusion was used to pull down another one in His-tag fusion (Figure 2A). His-tagged PCAF was clearly observed in beads fraction when incubated with MBP-PCAF but not in MBP alone (lanes 5 and 6) compared with a loading control (lane 1).

In order to analyze PCAF oligomeric state in cells, we performed a co-immunoprecipitation experiment using HA and Flag tags (Figure 2B). The full-length PCAF was tagged with either HA or Flag peptides at its N terminus. Flag affinity gel was used to bind Flag-PCAF. The HA-PCAF was readily detected when Flag-PCAF was co-expressed (lane 2), suggesting that over-expressed PCAF may self-associate to form oligomers in cells.

### PCAF dimers disrupted by mutations in interfaces

**Figure 3:**
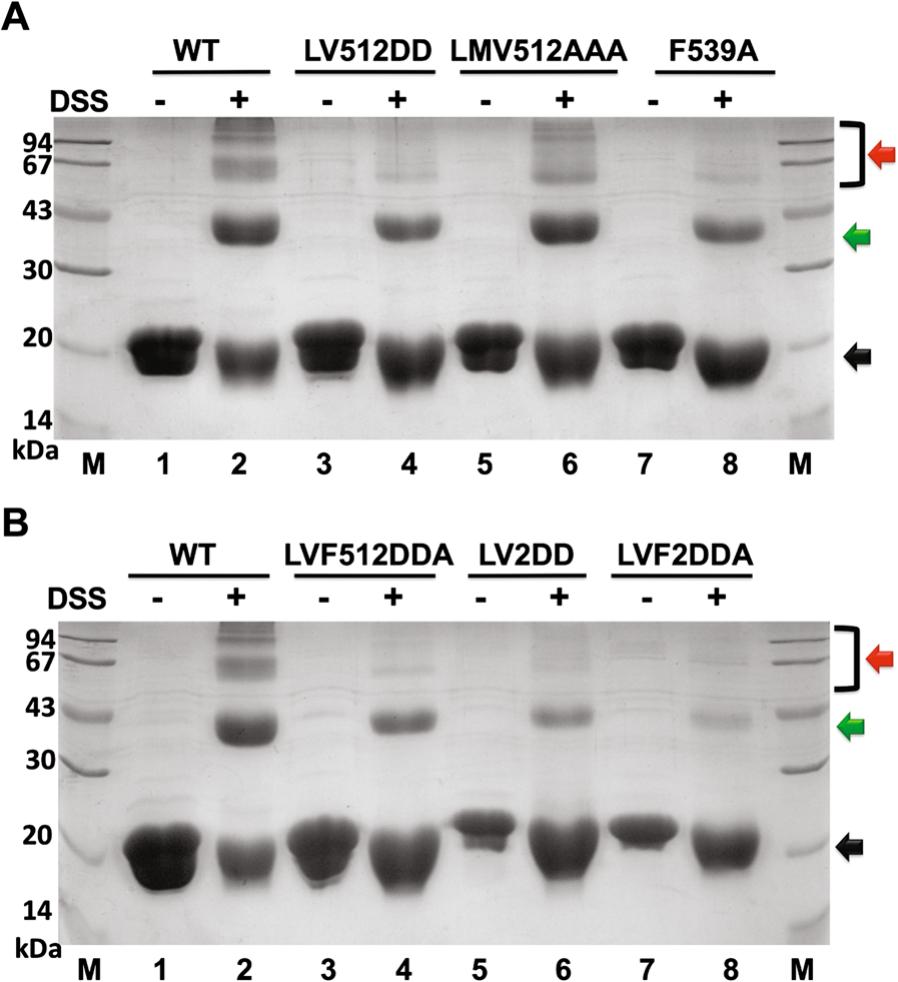
**PCAF HAT domain dimer detected by chemical cross-linking. A)** Chemical cross-linking of the full-length PCAF HAT domain (amino acids 493-658). WT represents the full-length wt PCAF HAT domain (lanes 1-2). LV512DD is a full-length HAT domain with L512D and V516D double mutations (lanes 3-4). LMV512AAA is a full-length HAT domain with L512A/M513A/V516A triple mutations (lanes 5-6). F539A is a full-length HAT domain with single F539 to alanine mutation (lanes 7-8). **B)** Cross-linking of a shortened PCAF HAT domain (amino acids 496-658) in comparison with a full-length PCAF HAT domain mutant. LVF512DDA is a combined mutant of LV512DD and F539A in the full-length PCAF HAT domain (lanes 3-4). LV2DD is a double mutation (L512D and V516D) in the shortened PCAF HAT domain (lanes 5-6) while LVF2DDA is a triple mutation (L512D, V516D and F539A) in the shortened HAT domain (lanes 7-8). The arrows indicate monomer (black), dimer (green) and higher oligomers (red). Lanes where DSS was added are indicated above the gel and M denotes a protein molecular weight marker.

To confirm the presence of the PCAF dimers in solution we then used a chemical cross-linking method (Figure 3). We observed a dominant dimer on SDS-PAGE after the PCAF HAT domain was incubated with a cross-linker disuccinimidyl suberate (DSS) (lane 2, indicated by green arrow). A ladder of higher molecular-weight oligomers was also appeared (lane 2, indicated by red arrow).

We continued to examine whether mutations in PCAF dimerization interfaces would affect its dimers’ formation. To disrupt the first interface, we generated a triple mutant LMV512AAA (Leu512, Met513 and Val516 to alanine) (Figure 1C). However, this mutant did not appear to affect the PCAF dimerization (Figure 3A, lane 6). In comparison, a mutant with the Leu512 and Val516 altered to aspartic acid (LV512DD) and Phe539 to alanine (F539A) did have small effects on the presence of PCAF dimer and higher oligomers (Figure 3A, lanes 4 and 8). An LV512DD and F539A combined mutant LVF512DDA was clearly less prone to form a dimer (Figure 3B, lane 4).

To disrupt the second interface, we removed the first three N terminal amino acids (amino acids 493-495) that are responsible for β-sheet extension (Figure 1D). However, the shortened PCAF HAT domain itself behaves as the wt PCAF in this experiment (Data not shown). The shortened PCAF HAT domain (amino acids 496-658) was then combined with mutations of the first interface studied above (Leu512 to Asp, Val516 to Asp and/or Phe539 to Ala) to generate two mutants (LV2DD and LVF2DDA). As expected, both mutations, in particular the LVF2DDA, clearly suppressed dimer formation of the HAT domain (Figure 3B, lanes 6 and 8).

These data support our crystallographic observation that both interfaces contribute to the dimeric formation of PCAF in our cross-linking experiment. However, the second interface may result from a crystal packing as suggested by our crystallographic analyses since the deletion mutant (amino acids 496-658) did not change its ability to form a dimer. It is important to note that our introduction of these mutations into PCAF did not affect enzymatic activity of PCAF HAT domain (Additional file 1: Figure S4).

### PCAF dimer detected by static light scattering

**Figure 4:**
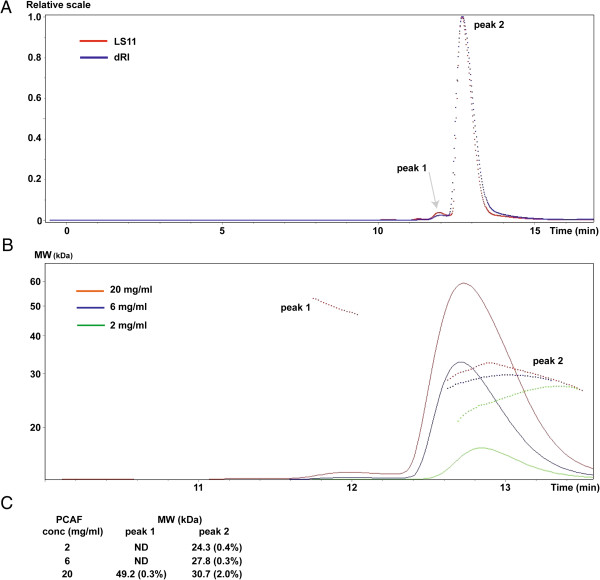
**Molecular weight of PCAF HAT domain determined by MALS. A)** A running profile of PCAF protein at concentration of 20 mg/ml. PCAF HAT domain was first run on a gel filtration column detected by light scattering (LS11, shown in red) and refractive index (dRI, shown in blue). The X axis is a running time taken from the HPLC. The LS11 (one of 18 light scattering detectors) and dRI intensities are aligned and scaled. A relative scale is shown as the Y axis. Peak 1 is small and thus highlighted by an arrow. **B)** The molecular weight of the PCAF HAT domain at different concentrations. The measurements are colored in green (2 mg/ml), blue (6 mg/ml) and red (20 mg/ml). The dotted lines represent averaged values for molecular weight calculated by 18 laser detectors at each time point. The X axis is the same HPLC running time as (A) and the Y axis indicates molecular weight. **C)** The molecular weights of two PCAF particles in solution. The number in parenthesis is an error rate, which was calculated using the measurements of all time points for each peak in panel B. ND is ‘Not able to Determine’ because of low signal.

To seek more evidence for presence of dimers in solution, we analyzed PCAF HAT domain using Multiangle static light scattering (MALS), which measures absolute molecular weight (MW) of a particle without any assumptions. A protein is first separated on a HPLC that is directly connected with detectors for measuring differential refractive index (dRI) and light scattering (LS). The theoretical MW of the HAT domain (amino acids 493-658) including N terminal His tag is 21.8 kDa. Here three different protein concentrations at 2, 6 and 20 mg/ml were used in this experiment (Figure 4). The peak 1 was analyzable only at 20 mg/ml, which was determined to be 49.2 kDa. The major portion of this protein remained in peak 2, which had the closest MW to PCAF HAT domain monomer at 2 mg/ml. The MW of peak 2 was 27.8 kDa at 6 mg/ml and 30.7 kDa at 20 mg/ml, suggesting that part of large particles from peak 1 was not separable. All these data indicate that PCAF HAT domain may remain in a dimer-monomer equilibrium with dominant monomeric species in solution.

## Discussion

Protein acetylation is well known for its role in epigenetic regulation of transcription and is also involved in translation, protein turnover, localization and quality control, thus linking acetylation to a variety of biological processes such as cell shape, migration and autophagy [32]. Moreover, PCAF/GCN5 family members have been implicated in carcinogenesis and drug targets for cancer therapy [33]. Marmorstein and his coworkers have extensively studied the structures and enzymatic mechanism of the catalytic domain and histone binding of these histone acetyltransferases [15,34]. Here in this report, our structural and biochemical analyses demonstrate that PCAF can exit as a dimer.

We solved the crystal structure of the PCAF HAT domain in two different dimeric states. One of these dimeric interfaces is large (more than 1500 Å^2^) created by several hydrogen bonds and hydrophobic contacts (Figure 1). The second interface is rather small, which may likely be generated during crystal packing (Additional file 1: Table S1). The crystals in this study were grown in 0.2 M lithium sulfate and 1.2 M ammoniumn sulfate at pH7.5, which were packed in the space group P4_3_. The same human PCAF HAT domain was crystallized with space group P6_4_ in a slightly different condition and no interfaces were found between its symmetric or asymmetric units [17]. We also crystallized human GCN5 HAT domain in a simple precipitant of 35% tacsimate pH 7.0 from Index screen kit (Hampton Research) and the crystal was packed in a space group I422 (a = b =129.2 and c = 179 Å) (Data not shown). Consistently, we found 4 GCN5 monomers in one asymmetric unit that form the same dimeric interfaces as those described above for PCAF (Additional file 1: Figure S5A for the major interface). The GCN5 dimer was well aligned with the PCAF with rmsd of 1.7 Å (Additional file 1: Figure S5B).

For comparison, we listed all crystal structures of PCAF/GCN5 homologues from tetrahymena, yeast and human (Additional file 1: Table S2). In one crystal form of a tetrahymena GCN5 HAT domain, a dimeric interface is also formed through anti-parallel helices H1 and H2 between two HAT domains in one asymmetric unit (Additional file 1: Figure S6A and S6B). Unfortunately, based on our PISA analysis, this interface is likely produced by a crystal packing (Additional file 1: Table S1). Consistently, two critical residues Met513 and Gln519 that are responsible for dimerization in human PCAF are replaced with two lysines in tetrahymena GCN5 (Additional file 1: Figure S6C).

In order to confirm whether the human PCAF HAT domain appears dimers in solution, we performed a series of experiments, including MBP pulldown, cross-linking and MALS (Figures 2, 3, 4). All these data supported that the PCAF HAT domain is able to form a dimer in solution. MALS experiment, however, indicated that the dimer forms only at low ratio because it could only be readily detected at higher protein concentration (Figure 4). The apparent MW was close to the theoretical one only at the lowest protein concentration, suggesting the larger MW was likely resulted of presence of the PCAF dimer.

Interestingly, the N terminal BRD domain of BRD2 protein has been found to be a dimer, allowing to significantly enhance the binding to histone H4 and aides with further recognition of the hypoacetylated H4K8 [35,36]. Indeed, the BRD domains of the PCAF/GCN5 family members also form dimers in crystallographic conditions (3D7C and 3GG3, Structural Genomics Consortium), which have relatively large interfaces (Additional file 1: Table S1). Even though their significant scores are 0, a synergistic effect of the HAT and BRD domains in dimer formation may possibly stabilize PCAF oligomeric state in vivo.

## Conclusions

**Figure 5:**
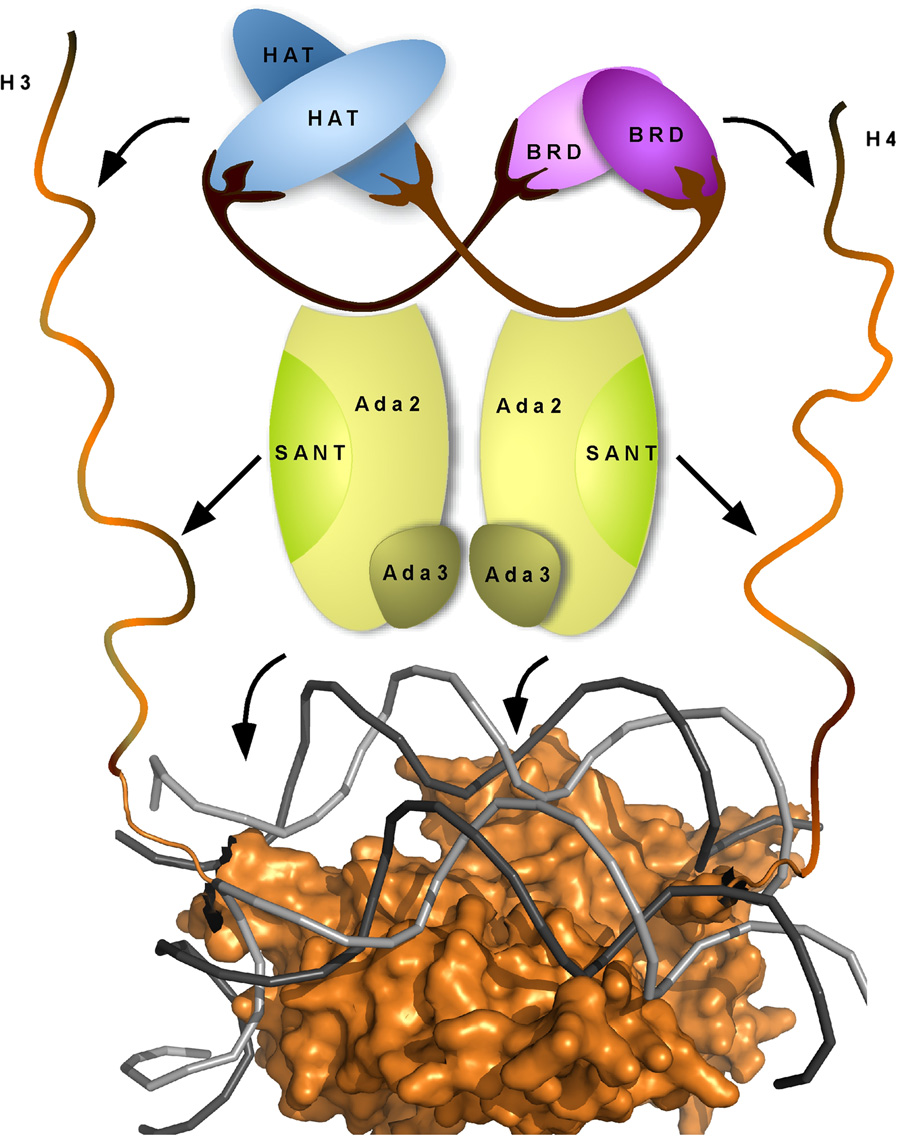
**A hypothetical model of core PCAF/GCN5 complex interacting with nuleosome for histone acetylation.** Shown in the bottom is a half nucleosome, which is made from the crystal structure solved by Luger et al [39]. An oligomer of histone H3 and H4 is wrapped with two double-strand DNA helices. The N terminal tails of H3 and H4 are extended manually. The three subunits of the PCAF/GCN5 core complex, including Ada2 and Ada3, are drawn schematically as a heterohexamer. The arrows indicate experimentally verified interactions between histone tails, DNA double strands and all individual domains.

Since PCAF/GCN5 always exist in megadalton complexes, one possibility is that PCAF dimerization may help to better associate with a nucleosome for efficient histone acetylation, which is depicted in our proposed model (Figure 5). PCAF/GCN5 attach to the nucleosomal DNA through Ada2 SWIM domain. PCAF/GCN5 bromodomain dimer may further dock the histone H4 through the H4K5 and H4K15 sites, which are acetylated before incorporated into the nucleosome [37]. The HAT domain dimer then recruits a histone H3 tail for acetylations at H3K14 and neighbor sites, including H3K9 and H3K18. The SANT domain of Ada2 may directly associate with the H3 and H4 tails and help to better position these for the acetylation process. Therefore, all these domains contribute to the overall acetylation level and specificity of histones even though their interactions with histones and DNA are extremely weak *per se*. Interestingly, the 3D reconstruction of complex SAGA has positioned GCN5 and other BRD-containing proteins in adjacent region, which may lead to a better association of SAGA and histone tails through multiple interactions [38].

In summary, our structural and biochemical studies suggest that the PCAF/GCN5 HAT domain can form a dimer in solution. We propose a model that this dimerization may be important for acetylations on specific sites of histones since multiple contacts may synergistically position PCAF/GCN5 megadalton complexes on the nucleosome. An important question whether PCAF/GCN5 are dimers in their functional complexes is currently under our investigations.

## Methods

### Protein expression and purification

Gene fragment encoding residues 493-658 of human PCAF HAT domain was amplified by PCR and subcloned into pET28a vector (Novagen) using *Nhe*I and *Xho*I restriction sites. Site-specific mutations and truncations were made using the modified Quikchange mutagenesis protocol [40]. The constructs were then used to express PCAF in *Echerichia. coli* stain BL21/DE3 (gold) using 0.25 mM isopropyl-ß-D-thiogalactopyranoside (IPTG) for 12 h at 25°C. PCAF proteins were purified by nickel affinity agarose (Qiagen) and Superdex 200 (GE Healthcare) according to the manufactures’ protocol. Purified proteins were concentrated down to ~20 mg/ml using centricons (Millipore) and stored at −80°C in a buffer of 20 mM Tris pH8.0, 100 mM NaCl, 300 mM ammonium acetate, 5 mM ß-mercaptoethanol (ß-ME).

### Protein crystallization

Initial crystals of the PCAF HAT domain were obtained by screening with JCSG plus (Qiagen) in one condition of 0.2 M lithium sulfate, 0.1 M Tris pH8.5 and 1.25 M ammonium sulfate. Further optimizations yielded the crystals in 0.2 M lithium sulfate, 0.1 M HEPES pH7.4, 1.2 M ammonium sulfate and 10 mM trimethylamine HCl. The crystals were harvested and snap-frozen in liquid nitrogen for diffraction data collection after a quick soaking in a buffer of 0.1 M HEPES pH7.4, 0.2 M lithium sulfate, 1.6 M ammonium sulfate and 15% isopropanol.

### Structural determination

Data were collected in Shanghai Synchrotron Radiation Facility (SSRF) and processed by HKL2000 [41]. The solution was found by molecular replacement using Phaser [42]. The model was rebuilt in Coot [43]. The final structure was refined and the model statistics (Table 1) were calculated using Phenix [44]. The buried surface area was calculated by PISA program [45]. All graphics for the various structures were produced using Pymol (DeLano Scientific LLC).

**Table 1:** Data collection and refinement statistics

**Space group**	**P 43**
Unit cell parameters (Å)	a = b = 65.57 Å, c = 187.62 Å
Resolution (Å)	50.0-2.31 (2.35-2.31)^†^
Reflections (Total/Unique)	344112/32820
^††^I/σ (I)	45.7 (3.1)
*R_sym_ (%)	11.8 (55.1)
Completeness (%)	94.9 (62.5)
Refinement	
Resolution (Å)	45.26-2.31 (2.37-2.31)
Reflections (after 2**σ** cutoff)	32806 (2376)
Completeness (after 2**σ** cutoff) (%)	95.0
^‡^R (%)	22.8 (28.3)
^**^R_free_ (%)	26.5 (33.7)
Model quality	
^§^RMSD bond length (Å)	0.012
RMSD bond angles (°)	1.261
Overall B-factor (Å^2^)	80.2
Ramachadran plot	98.9% (favorite)
	1.1% (allowed)
	0% (disallowed)
***Number of total atoms	5296
Protein atoms	5104
Ligand atoms	192
H2O	27

†The data for the highest resolution shell are shown in parenthesis. *Rsym = S|I- < I > |/SI, where I is the observed intensity, <I > is the statistically weighted average intensity of multiple observations of symmetry-related reflections. ‡R = S||Fo|–|Fc||/S|Fo|, where Fo and Fc are observed and calculated structure factor amplitudes, respectively. **R_free_ is calculated for a randomly chosen 10% of reflections. §RMSD–root mean square deviation. ††I/**σ**(I)–ratio of mean intensity to a mean standard deviation of intensity. *** Number of protein atoms and nucleic acid atoms–the ordered region.

### Maltose binding protein (MBP) pulldown

MBP-PCAF HAT domain (amino acids 493-658) fusion protein and MBP alone were expressed in pMBP-c, a modified vector from pMAL-c2x (NEB) with convenient restriction and thrombin cleavage sites. The fusion protein and MBP were purified using amylose agarose (New England Biolab). An extra C terminus of the MBP alone (~30 amino acids) using this empty pMBP-c vector was sensitive to protease cleavage and showed two bands in our SDS-PAGE. All bound maltose was efficiently removed by running these proteins on a gel filtration Superdex 200 (GE Healthcare) using a high salt buffer of 30 mM Tris pH8.0, 2 M NaCl, 5 mM ß-ME and 3 mM EDTA. The MBP or MBP-PCAF proteins (60 μg) were then rebound with 10 μl new amylose agarose beads and 60 μg His-tagged PCAF (amino acids 493-658 and 493-832) in a binding buffer of 50 mM Tris pH8.0, 150 mM sodium chloride, 5 mM ß-ME and 3 mM EDTA for 2 h at 4°C. After 3 washes using the binding buffer added with 0.03% Triton X-100, the agarose beads were collected and the bound proteins were subjected to a regular 15% SDS-PAGE. The gel was stained with coomassie blue.

### Co-immunoprecipitation

Plasmid pCI-Flag PCAF of Flag-tagged full-length human PCAF was purchased from Addgene [13]. The construct expressing HA-PCAF was built on pLV-nHA vector using EcoRI and XbaI sites and confirmed by DNA sequencing. Cells 293 T were cultured in DMEM and 10% fetal bovine serum (Gibco) with 100 μg/ml penicillin and streptomycin. Co-transfection of both tagged PCAF expression plasmids (8 μg each) was carried out using standard calcium phosphate method. Cells were harvested after 48 h and lysed in a lysis buffer (50 mM Tris–HCl pH7.4, 150 mM NaCl, 1 mM PMSF, 10 μg/ml leupeptin, 2 μg/ml pepstatin, 1 mM EDTA and 1% Triton X-100). The lysed supernatants were incubated with 20 μl anti-Flag M2 affinity gel (Sigma) for 4 h. The Flag gel was then harvested and washed using the same lysis buffer and directly mixed with SDS sample buffer. The mixture was resolved by 10% SDS-PAGE. The gel was blotted to nitrocellulose membrane and further detected using anti-Flag or anti-HA antibodies (Sigma).

### Cross-linking

Proteins (wt or mutated HAT domains) were exchanged to a non-amine buffer (50 mM HEPES pH7.5, 50 mM sodium sulfate) by dialysis. Protein cross-linking was carried out using 50 mM disuccinimidyl suberate (DSS) dissolved in pure dimethyl sulfoxide (DMSO). DSS at 5X molar concentration was directly added to 10 μl of 1 mg/ml PCAF proteins. The reactions were incubated for 1 h at room temperature and quenched by adding 0.3 M Tris pH7.5 to reach a final concentration of 30 mM. The reactions were then analyzed using regular 12% SDS-PAGE and stained with coomassie blue.

### Multiangle laser light scattering (MALS)

The protein prep of the PCAF HAT domain at 2-20 mg/ml was first resolved on a size exclusion column (WTC-010S5, 5 μm silica beads, 7.8 × 300 mm) in a buffer of 50 mM phosphate buffer at pH 7.0 and 150 mM NaCl at 35°C. The HPLC was run on a LabAlliance series 1500 isocratic system at a flow rate of 0.5 ml/min. Data were then collected on a DAWN HELEOS II laser photometer at an emission of 658 nm (Wyatt, USA). Molecular mass was calculated using ASTRA V (Wyatt, USA).

## Abbreviations

Ada: Alteration/deficiency in activation; ATAC: Ada Two-A containing complex; BRD: Bromodomain; DMSO: Dimethyl sulfoxide; dRI: Differential refractive index; DSS: Disuccinimidyl suberate; HAT: Histone acetyltransferase; GCN5: General control nonderepressible 5; IPTG: Isopropyl-ß-D-thiogalactopyranoside; MBP: Maltose binding protein; MALS: Multiangle light scattering; MW: Molecular weight; PCAF: p300/CBP associated factor; rmsd: Root mean square deviation; wt: Wild-type.

## Competing interests

The authors declare that they have no competing interests.

## Authors’ contributions

SS, JL and JY crystallized this protein and did further biochemical experiments. YC collected data and solved this structure. HH and XL did co-IP experiment. AH, KJ and RC formulated the designs of these studies. AH and JH managed these experiments. AH analyzed data and wrote the manuscript. JSD revised this manuscript. All authors read and approved the final manuscript.

## Supplementary Material

Additional file 1: Figure S1 Two dimeric PCAF HAT structures in one asymmetric unit. **Figure S2**. Structural comparison of PCAF HAT domain with its homologues. **Figure S3**. Electron density map of two dimeric interfaces. **Figure S4**. The PCAF mutants are enzymatically active as wt PCAF. **Figure S5**. Human GCN5 HAT domain crystallized in a dimeric state. **Figure S6**. Dimeric structure of tetrahymena GCN5. **Table S1**. PISA analyses of the dimeric interfaces of PCAF/GCN5 crystal structures. **Table S2**. Crystal structures of PCAF/GCN5 homologues that have currently been solved. Click here for file

## Accession number

Coordinates and structure factors have been deposited in the Protein Data Bank with accession number 4NSQ.
