# Supplementary material for: Dimeric structure of p300/CBP associated factor
Source: BMC Struct Biol. 2014 Jan 14;14:2. doi: 10.1186/1472-6807-14-2 (PMC3897949; doi:10.1186/1472-6807-14-2)
Supplement: Additional file 1: Figure S1 — Two dimeric PCAF HAT structures in one asymmetric unit. Figure S2. Structural comparison of PCAF HAT domain with its homologues. Figure S3. Electron density map of two dimeric interfaces. Figure S4. The PCAF mutants are enzymatically active as wt PCAF. Figure S5. Human GCN5 HAT domain crystallized in a dimeric state. Figure S6. Dimeric structure of tetrahymena GCN5. Table S1. PISA analyses of the dimeric interfaces of PCAF/GCN5 crystal structures. Table S2. Crystal structures of PCAF/GCN5 homologues that have currently been solved. [file 1472-6807-14-2-S1.doc]

Supplementary materials for Shi et al

**Dimeric structure of p300/CBP associated factor**

**Index**

Figure S1. Two dimeric PCAF HAT structures in one asymmetric unit.

Figure S2. Structural comparison of PCAF HAT domain with its homologues.

Figure S3. Electron density map of two dimeric interfaces.

Figure S4. The PCAF mutants are enzymatically active as wt PCAF.

Figure S5. Human GCN5 HAT domain crystallized in a dimeric state.

Figure S6. Dimeric structure of tetrahymena GCN5.

Table S1. PISA analyses of the dimeric interfaces of PCAF/GCN5 crystal structures.

Table S2. Crystal structures of PCAF/GCN5 homologues that have currently been solved.

Figure S1


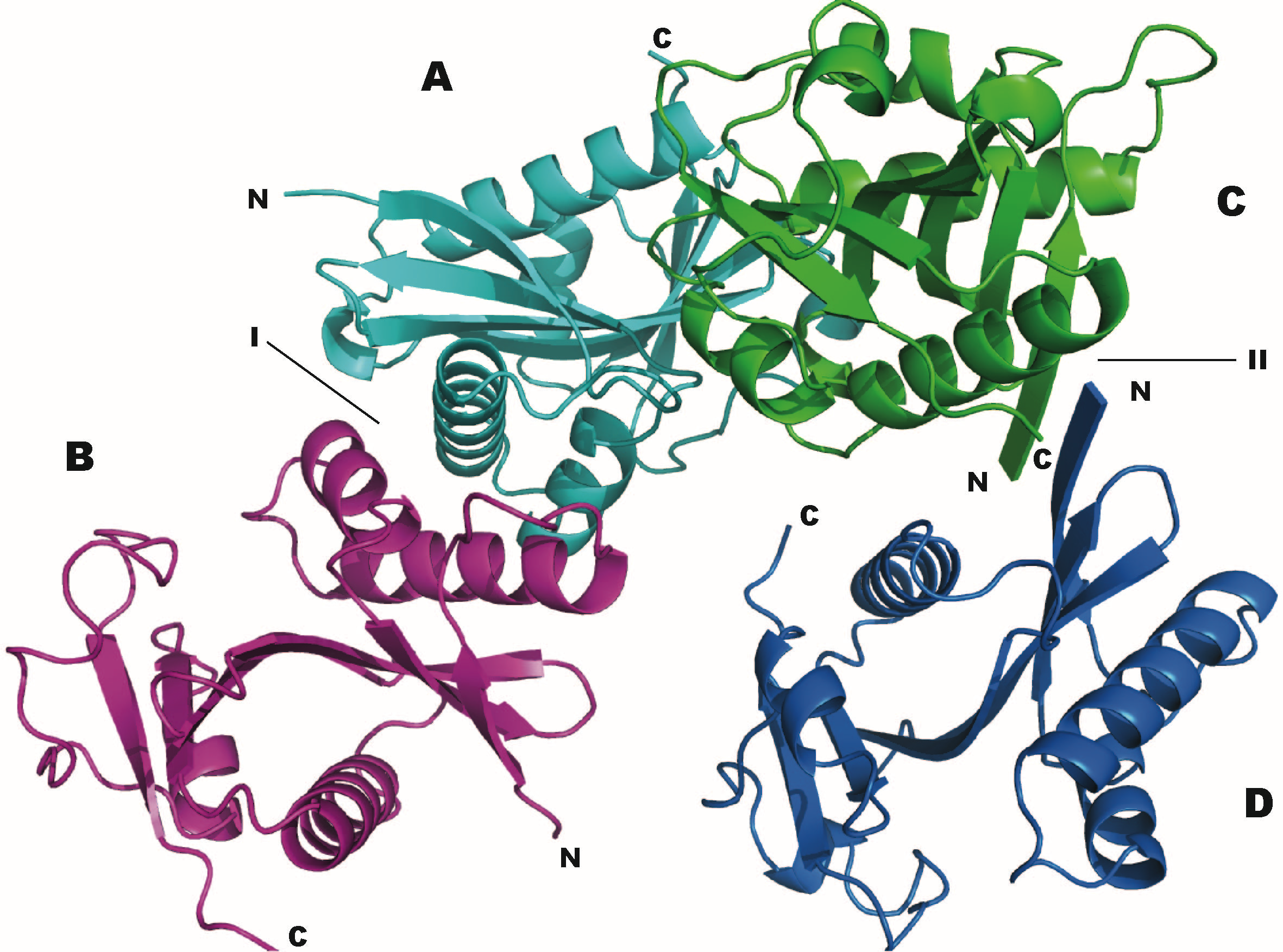


Figure S1. Two dimeric PCAF HAT structures in one asymmetric unit.

The four PCAF HAT domains (A, B, C and D) are packed into one asymmetric unit. The PCAF is colored by cyan, magenta, green and blue, respectively. Ends of each PCAF peptide are labeled with N (Amino terminus) and C (Carboxyl terminus). Cofactors acetyl-CoA are omitted for clarity. The two dimeric interfaces are marked by I and II.

Figure S2


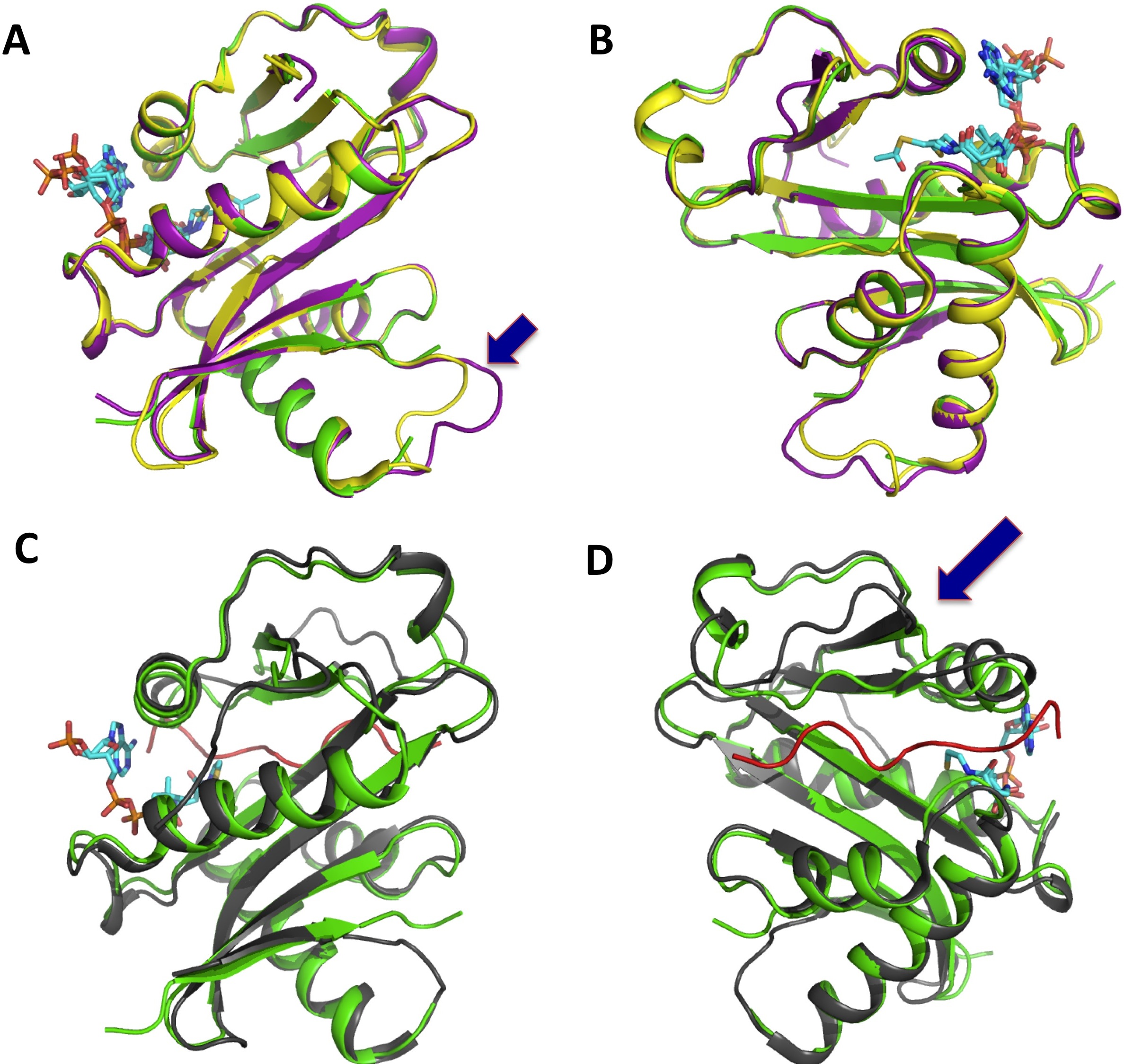


Figure S2. Structural comparison of PCAF HAT domain with its homologues.

A) Alignment of PCAF structure reported in this study (green) to the ligand-free PCAF (purple) and GCN5 (yellow) structures that were previously solved [3, 4]; B) Flipped-over view of A; C) Alignment of PCAF structure from this study (green) to tetrahymena GCN5 structure (grey) with a bound histone (red) [5]; D) Flipped-over view of C. All structures are presented in ribbons with the cofactor acetyl-CoA shown in sticks. The bound tetrahymena GCN5 is shown in grey with histone tail colored in red. Blue arrows indicate the loop 1 of our PCAF structure (A) and a large shift of the loop in active site when histone H3 tail is bound (D).

Figure S3

A B


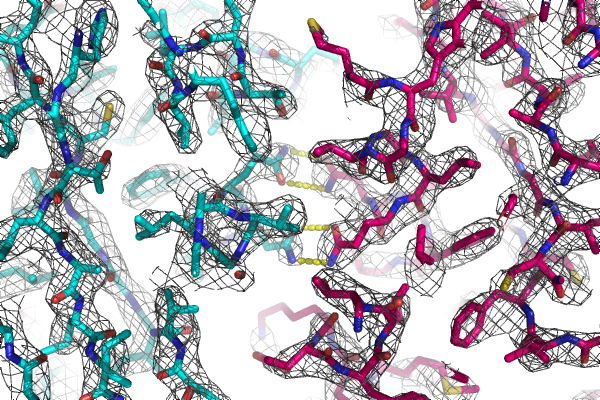

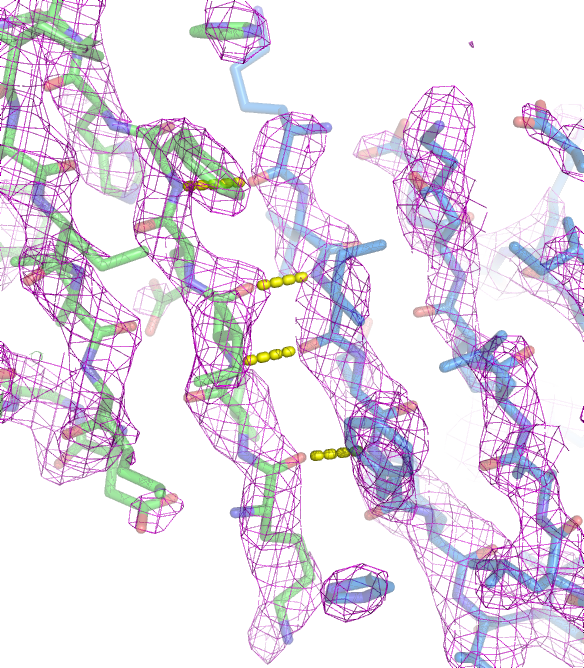


Figure S3. Electron density map of two dimeric interfaces.

Electron density maps are shown for parts of PCAF dimeric interfaces (A for interface I and B for interface II). PCAF monomers are shown in stick models. Electron density maps (2Fo-Fc map) are drawn in grey (A) and magenta (B). The maps are contoured at 1.5σ. The hydrogen bonds are highlighted in dashed lines as Figure 1.

Figure S4


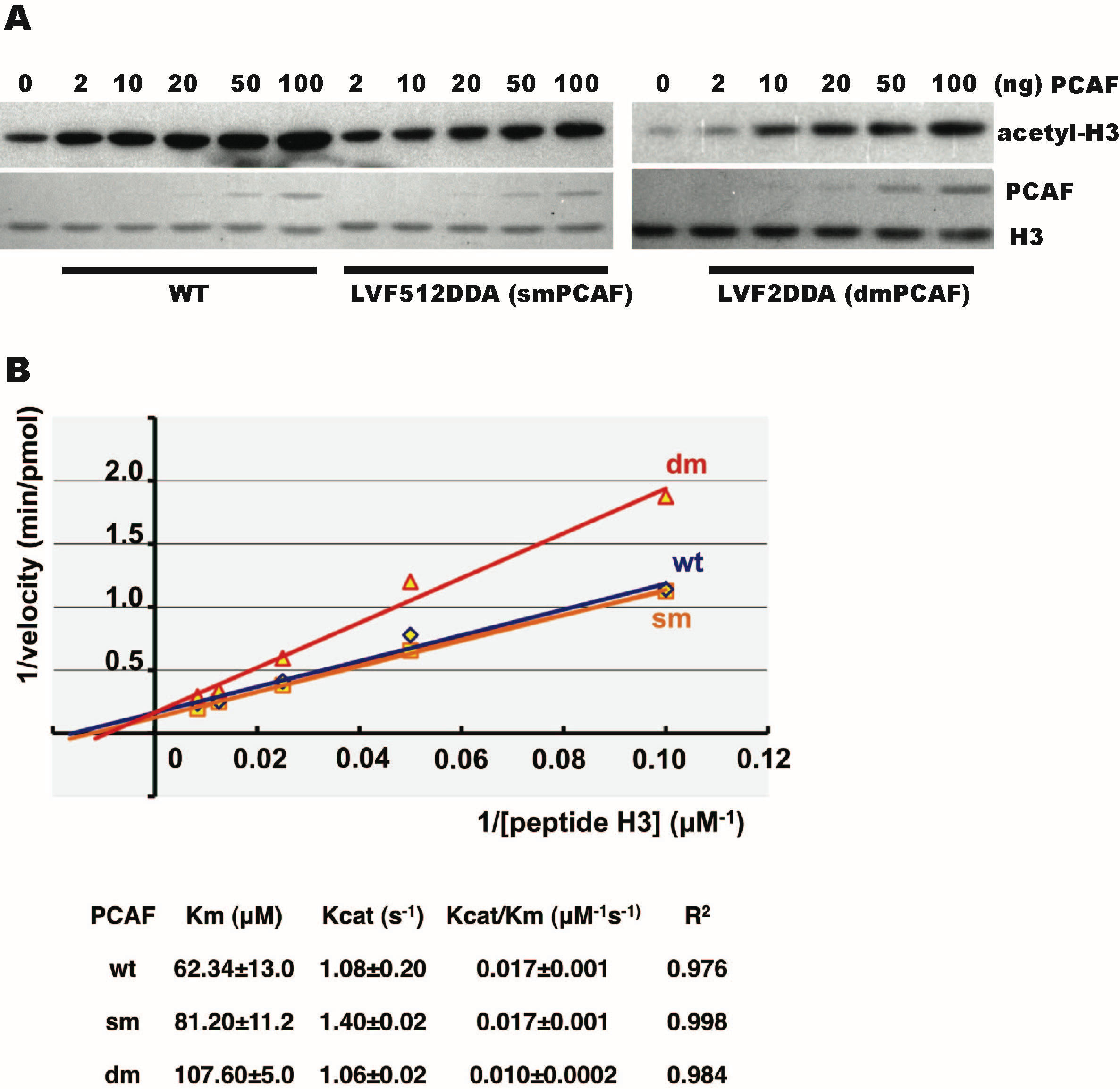


Figure S4. The PCAF mutants are enzymatically active as wt PCAF.

A) Enzymatic analysis of PCAF using purified and refolded histone H3 as a substrate. *Xenopus laevis* histone protein H3 expression constructs were kindly provided by Dr. Karolin Luger (Colorado State University). Histone H3 was purified from inclusion bodies and refolded according to Luger’s protocol [1]. The acetylation reaction with 0-100 ng PCAF (labeled on the top) was set up with 100 ng H3 in buffer of 50 mM Tris pH8.5,10 mM butyric acid,10 % glycerol and 0.1 mM EDTA for 30 min at 37 °C according to Chakravarti *et al* [2]. The mixtures were separated on 15 % SDS-PAGE and western blot was performed using anti-acetyl-K14 histone H3 antibody (06-599, Millipore). B) PCAF enzymatic kinetics was measured using a fluorescence based HAT Assay kit (Active Motif, USA), following the manufacture's instruction. Briefly, 50 ng of purified wt or mutant PCAF HAT domains were mixed in a total volume of 25 μl with a synthetic peptide substrate of the first 23 amino acids of histone H3. The reactions were incubated for 60 min at room temperature and stopped by a stop solution provided in the kit. After the addition of a developing solution for 15 min at room temperature the fluorescence was measured at 450 nm (excited at 360 nm) in a POLARstar Omega plate reader (BMG Labtech). At least two independent experiments were performed and fitted into Lineweaver-Burk plot using the root-mean-least square equation 1/V=(K_m_/V_max_) (1/[S])+1/V_max_.

Figure S5

A B


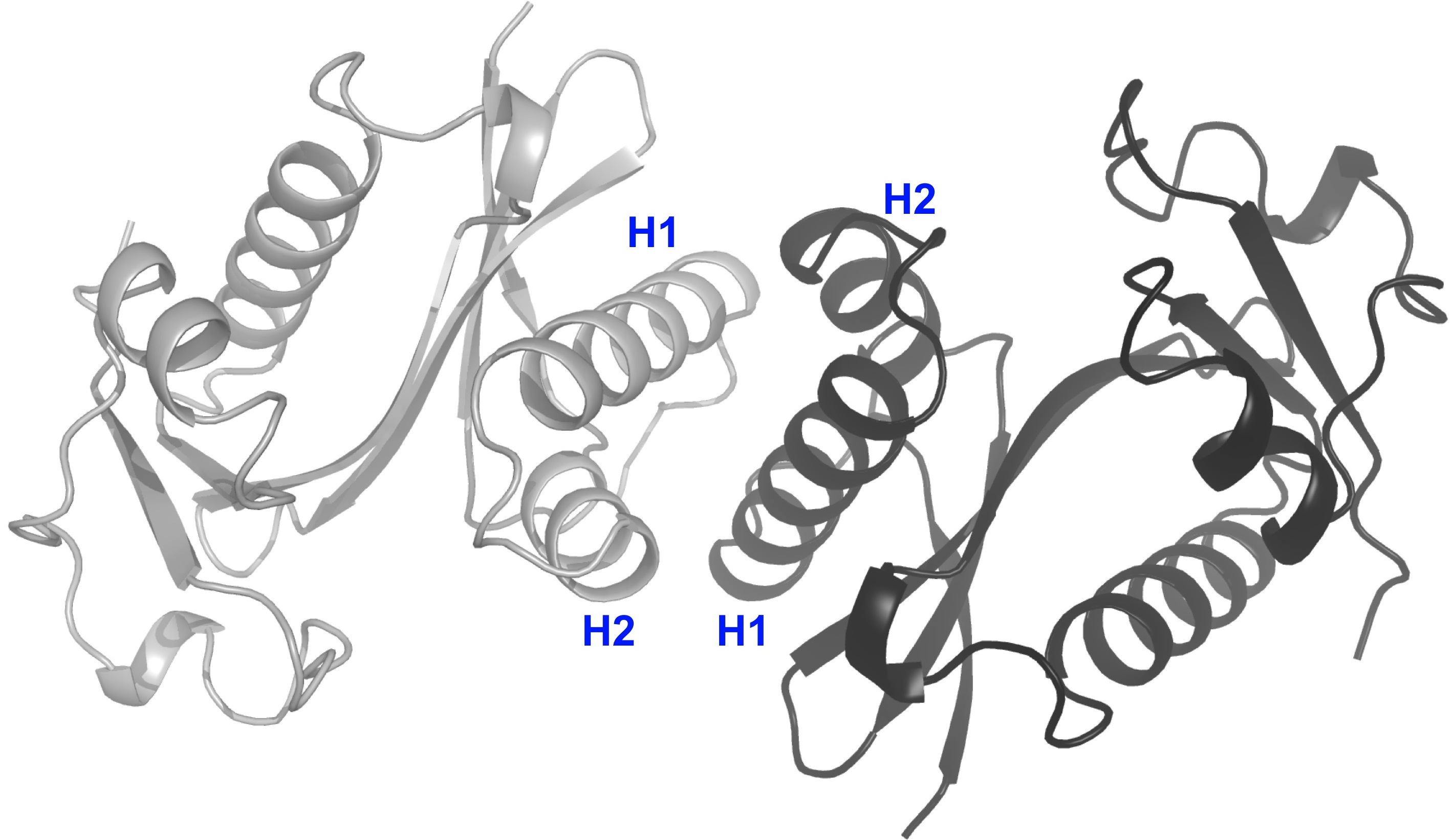

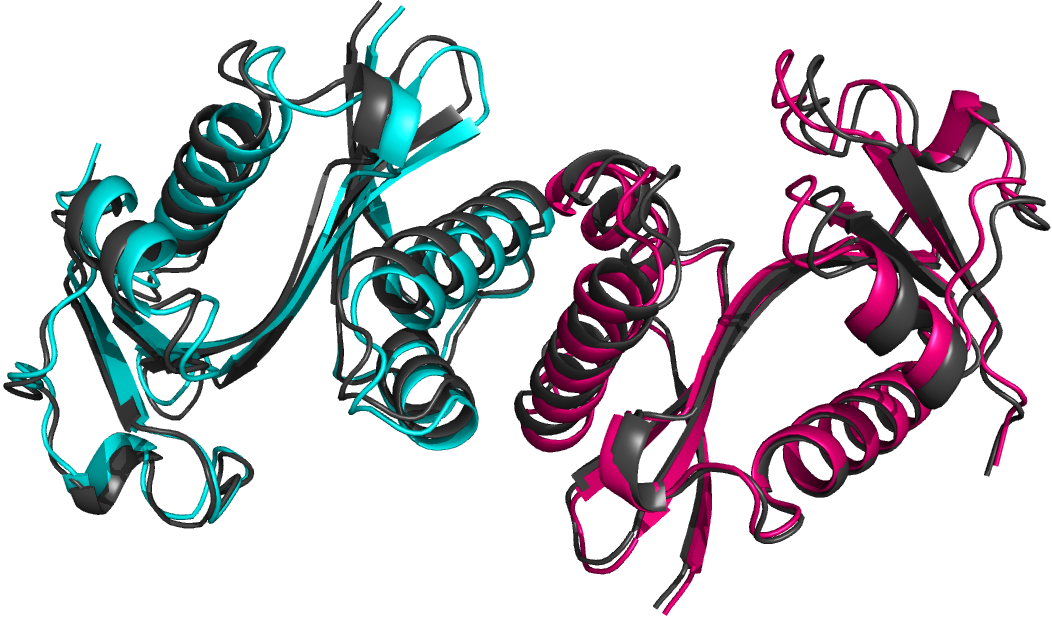


Figure S5. Human GCN5 HAT domain crystallized in a dimeric state.

Human GCN5 (hGCN5) fragment (amino acids 493-662) was cloned into pET28a and expressed in BL21/DE3 Gold. The protein was purified using nickle affinity agarose and gel filtration. Protein sample was concentrated down to 6 mg/ml and screened for crystals at room temperature. Initial crystals were found in condition 28 that contained only 35% Tacsimate. The diffraction data was collected at 3.91 Å (space group is I422 and cell 129.189, 129.189, 179.132). The structure was solved as described in method section but without further refinement. A) One of the major dimer interfaces formed by a 4-helical bundle. B) Structural alignment of major dimers of hGCN5 and PCAF. Two GCN5 monomers are colored in grey and dark grey while two PCAF monomers are colored in cyan and magenta.

Figure S6

A


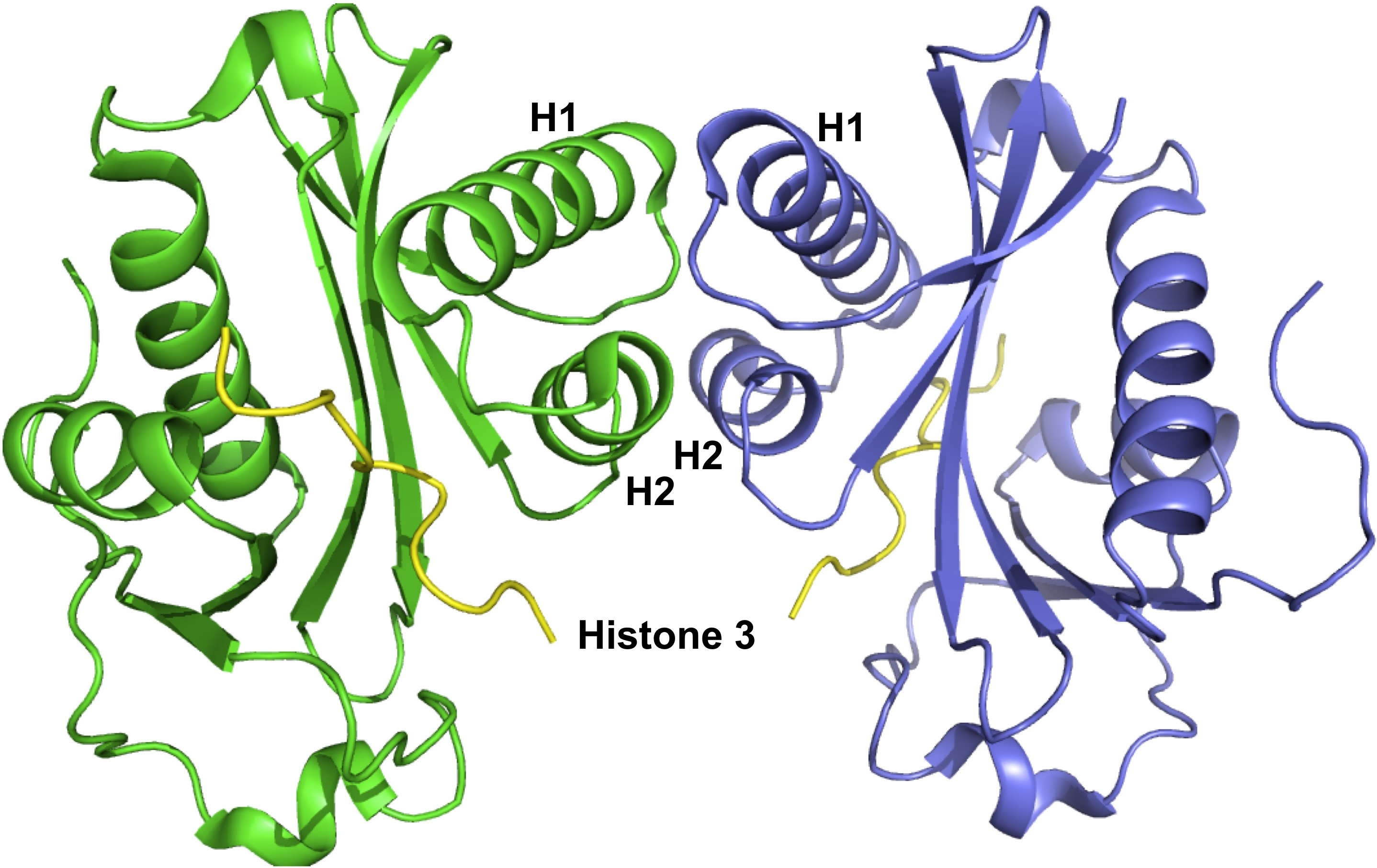


B


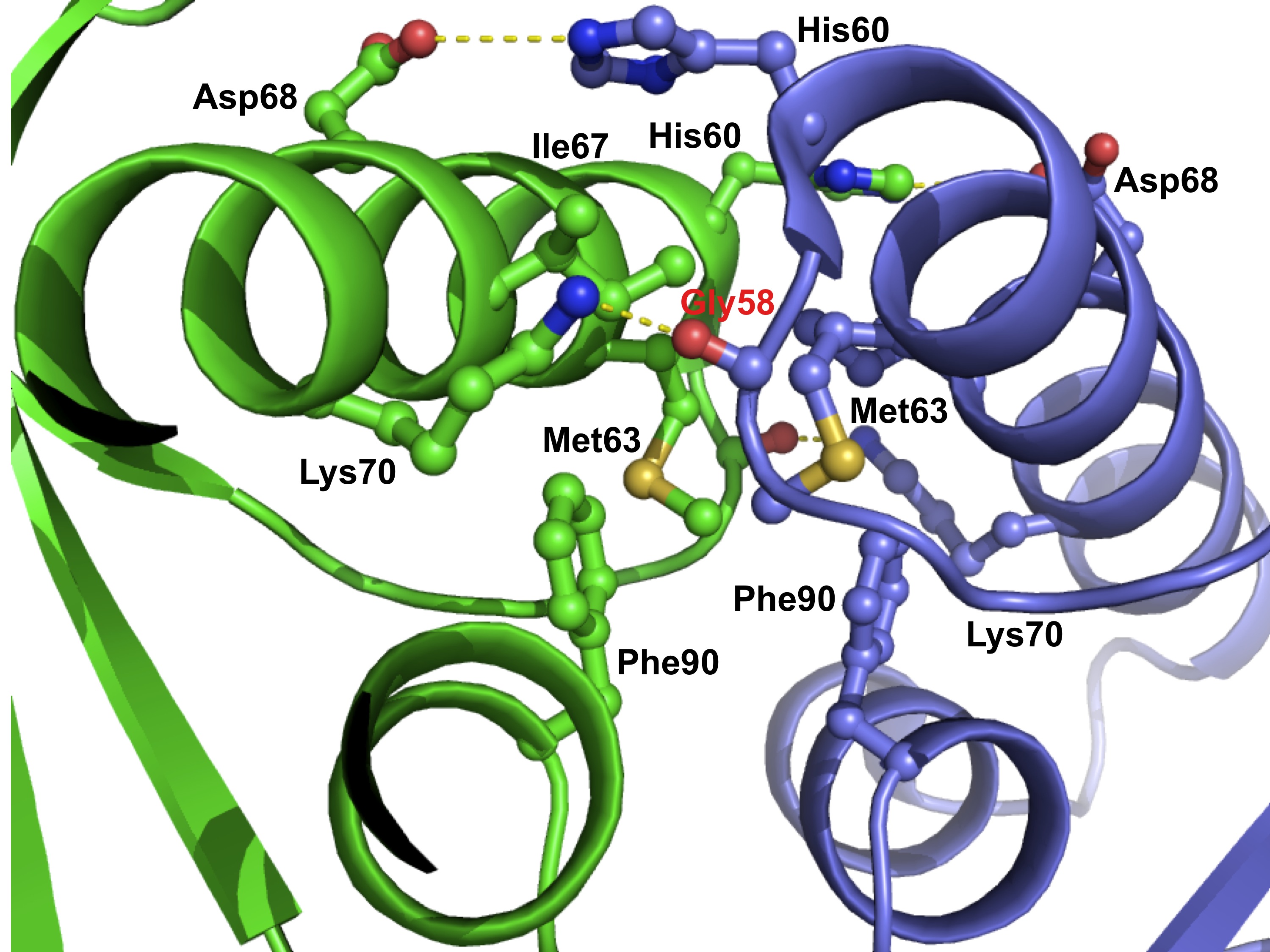


C


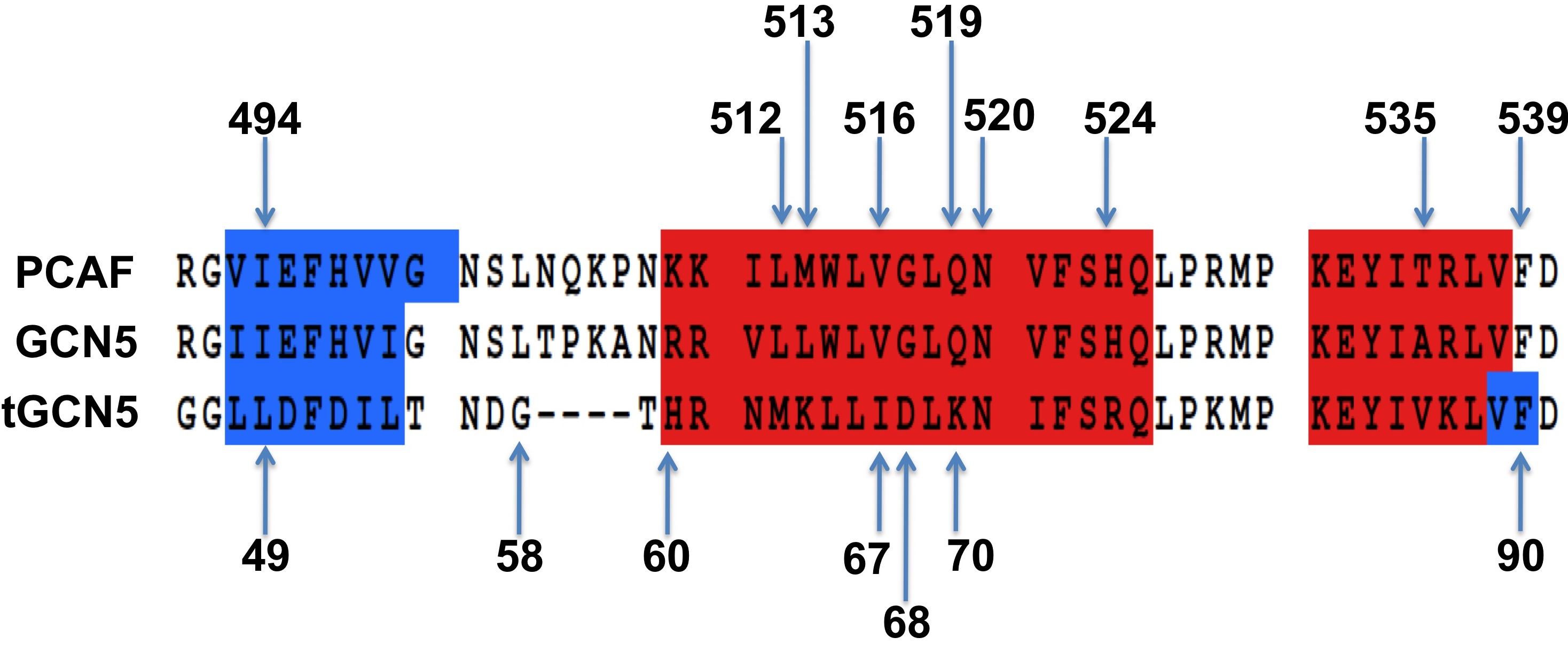


Figure S6. Dimeric structure of tetrahymena GCN5.

The tetrahymena GCN5 (tGCN5) structures with acetyl-CoA, p53 peptide and histone H3 and H4 tails were solved by Marmorstein and his colleagues [5-7]. A) Two tetrahymena GCN5/H3 complexes form an anti-parallel 4-helical bundle using helices H1 and H2 through crystal packing or within asymmetric unit. The acetyl-CoA has been omitted for better clarity. Histone H3 tails in bound state are colored in yellow. B) Detailed interface of the GCN5 dimer. The buried surface area is 1065 Å^2^. Labeled are amino acids that are involved in close contacts. Four hydrogen bonds are formed within amino acids pairs of His60/Asp68 and Gly58/Lys70. The hydrophobic interactions are found in two identical groups between Met63, Ile67 and Phe90. C) Conservation of the key residues for dimerization between human PCAF/GCN5 and tGCN5. The alignment was performed in PRALINE server [8]. The β-strand is colored in blue and α-helices in red. The residues are numbered according to the original sequences of human PCAF (top) and tGCN5 (bottom).

Table S1

PISA analyses of the dimeric interfaces of PCAF/GCN5 crystal structures.


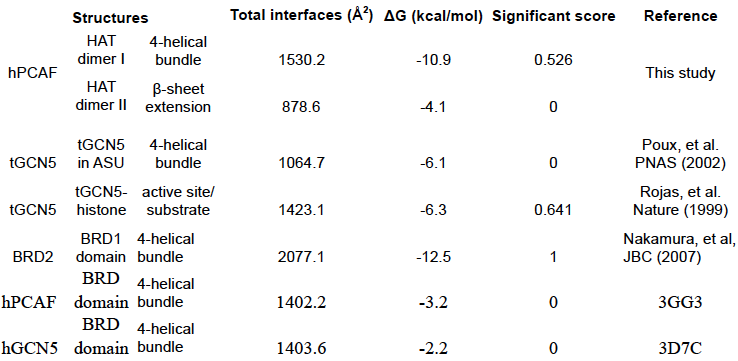


Human PCAF (hPCAF) reported in this study was analyzed in two different dimeric formats (dimers I and II). In comparison, tetrahymena GCN5 (tGCN5) had two monomers in one asymmetric unit and formed a 4-helical bundle interface [6], which could be also found between its symmetric mates of earlier crystal structure of tGCN5 and histone H3 tail complex [5]. However, its significant score is 0, suggesting it was likely resulted from crystal packing. As a positive control, the interface of tGCN5 and histone H3 tail, where the histone is bound into tGCN5 active site, had good size of interface area and significant score.

Table S2

Crystal structures of PCAF/GCN5 homologues that have currently been solved.


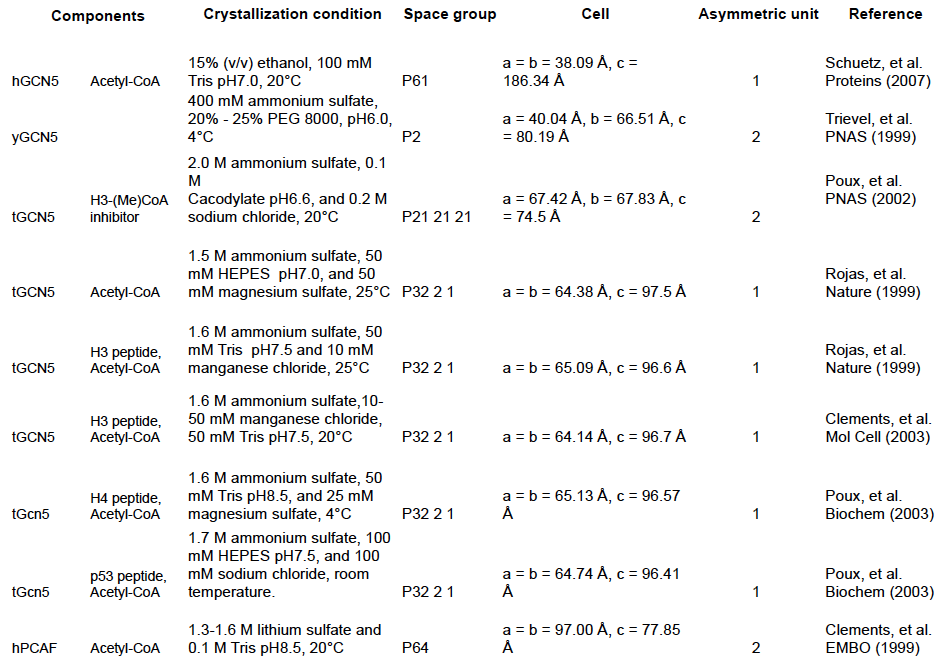


These crystal structures are listed from publications by Schuetz et al [9], Trievel et al [10], Poux et al [6], Rojas et al [5], Clements et al [11], Poux et al [7] and Clements et al [3]. Asymmetric unit is a number of molecules in one asymmetric unit.

**Reference**

1. Luger K, Rechsteiner TJ, Flaus AJ, Waye MM, Richmond TJ: **Characterization of nucleosome core particles containing histone proteins made in bacteria**. *J Mol Biol* 1997, **272**(3):301-311.

2. Chakravarti D, Ogryzko V, Kao HY, Nash A, Chen H, Nakatani Y, Evans RM: **A viral mechanism for inhibition of p300 and PCAF acetyltransferase activity**. *Cell* 1999, **96**(3):393-403.

3. Clements A, Rojas JR, Trievel RC, Wang L, Berger SL, Marmorstein R: **Crystal structure of the histone acetyltransferase domain of the human PCAF transcriptional regulator bound to coenzyme A**. *EMBO J* 1999, **18**(13):3521-3532.

4. Schuetz A, Bernstein G, Dong A, Antoshenko T, Wu H, Loppnau P, Bochkarev A, Plotnikov AN: **Crystal structure of a binary complex between human GCN5 histone acetyltransferase domain and acetyl coenzyme A**. *Proteins* 2007, **68**(1):403-407.

5. Rojas JR, Trievel RC, Zhou J, Mo Y, Li X, Berger SL, Allis CD, Marmorstein R: **Structure of Tetrahymena GCN5 bound to coenzyme A and a histone H3 peptide**. *Nature* 1999, **401**(6748):93-98.

6. Poux AN, Cebrat M, Kim CM, Cole PA, Marmorstein R: **Structure of the GCN5 histone acetyltransferase bound to a bisubstrate inhibitor**. *Proc Natl Acad Sci U S A* 2002, **99**(22):14065-14070.

7. Poux AN, Marmorstein R: **Molecular basis for Gcn5/PCAF histone acetyltransferase selectivity for histone and nonhistone substrates**. *Biochemistry* 2003, **42**(49):14366-14374.

8. Simossis VA, Heringa J: **PRALINE: a multiple sequence alignment toolbox that integrates homology-extended and secondary structure information**. *Nucleic Acids Res* 2005, **33**(Web Server issue):W289-294.

9. Schuetz A, Bernstein G, Dong A, Antoshenko T, Wu H, Loppnau P, Bochkarev A, AN. P: **Crystal structure of a binary complex between human GCN5 histone acetyltransferase domain and acetyl coenzyme A.** *Proteins* 2007, **68**(1):403-407.

10. Trievel RC, Rojas JR, Sterner DE, Venkataramani RN, Wang L, Zhou J, Allis CD, Berger SL, Marmorstein R: **Crystal structure and mechanism of histone acetylation of the yeast GCN5 transcriptional coactivator**. *Proc Natl Acad Sci U S A* 1999, **96**(16):8931-8936.

11. Clements A, Poux AN, Lo WS, Pillus L, Berger SL, Marmorstein R: **Structural basis for histone and phosphohistone binding by the GCN5 histone acetyltransferase**. *Mol Cell* 2003, **12**(2):461-473.
